# Changes in Plasma Ghrelin and Serum Leptin Levels after Cisplatin-Based Transcatheter Arterial Infusion Chemotherapy for Hepatocellular Carcinoma

**DOI:** 10.1155/2013/415450

**Published:** 2013-03-07

**Authors:** Tomoaki Matsumura, Makoto Arai, Masaharu Yoshikawa, Kentaro Sudo, Kazuyoshi Nakamura, Tatsuro Katsuno, Fumihiko Kanai, Taketo Yamaguchi, Osamu Yokosuka

**Affiliations:** ^1^Department of Gastroenterology and Nephrology, Graduate School of Medicine, Chiba University, Chiba City 260-8670, Japan; ^2^Department of Endoscopic Diagnostics and Therapeutics, Chiba University Hospital, Chiba City 260-8670, Japan; ^3^Hongo Avenue Medical Clinics, Chiba City 262-0033, Japan; ^4^Department of Gastroenterology, Chiba Cancer Center, Chiba City 260-8717, Japan

## Abstract

*Background and Objective.* Cisplatin-based chemotherapy is widely recognized to cause severe gastrointestinal disorders like nausea, vomiting, and appetite loss. The aim of this study was to assess whether cisplatin-based transcatheter arterial infusion (TAI) chemotherapy reduces plasma ghrelin levels and food intake in hepatocellular carcinoma (HCC) patients. *Methods.* Seventeen patients with HCC who underwent cisplatin-based TAI chemotherapy (80–100 mg/body) were enrolled in this study. Changes in peptide hormones, including ghrelin and leptin, as well as cytokines, were measured before and after chemotherapy. Appetite was evaluated by visual analog scale (VAS) and food intake was scored by eleven stages (0–10). *Results.* Appetite and food intake were significantly decreased after chemotherapy (*P* < 0.05). Plasma acylated ghrelin levels before therapy and at day 1, day 7, and day 14 after chemotherapy were 10.4 ± 7.2, 4.7 ± 4.7, 11.7 ± 8.9, and 9.3 ± 6.6 fmol/mL, respectively. The level on day 1 was decreased significantly (*P* < 0.05). In contrast, the levels of leptin, granulocyte colony-stimulating factor (G-CSF), and monocyte chemotactic protein-1 (MCP-1) on day 1 were increased significantly (*P* < 0.05). *Conclusions.* TAI for HCC reduced plasma acylated ghrelin levels, appetite, and food intake significantly. In addition, it increased serum leptin levels.

## 1. Introduction

Cisplatin-based chemotherapy is widely recognized to cause severe gastrointestinal disorders like nausea, vomiting, and appetite loss. The acute phase of cisplatin-induced gastrointestinal disorders involves increased serotonin (5-hydroxytryptamine (5-HT)) secretion from enterochromaffin cells [1]. Consequently, the 5-HT3-receptor antagonist was developed and is widely used for patients who undergo chemotherapy. However, many patients still suffer from gastrointestinal disorders.

Ghrelin is a 28-amino acid peptide found in the stomach. It is an endogenous ligand for growth-hormone secretagogue receptors [2]. Ghrelin is known to have an intense appetite-enhancing effect in addition to the growth-hormone-secretion-promoting effect [3]. Ghrelin is the only hormone that exhibits an orexigenic effect following peripheral administration [4]. In addition, ghrelin exhibits a variety of actions including stimulation of growth hormone (GH) secretion, gastric motility and gastric acid secretion, and induction of positive energy balance [5, 6]. Recently, it has been reported that ghrelin can greatly alleviate the behaviors associated with chemotherapy-induced dyspepsia in rodents [7]. In rats, administration of cisplatin resulted in marked decrease in plasma ghrelin and exogenously administered ghrelin improved cisplatin-induced reduction of food intake [8]. In humans, very recently, Hiura et al. reported that the administration of ghrelin during chemotherapy stimulated food intake and minimized adverse events [9]. However, the effect of exogenous ghrelin on the efficacy of chemotherapy in humans has rarely been investigated.

The aim of this study was to assess whether transcatheter arterial infusion (TAI) chemotherapy reduces plasma ghrelin levels and clarify the relationship between peptide hormones and foodintake activity in hepatocellular carcinoma (HCC) patients.

## 2. Materials and Methods

### 2.1. Patients

Seventeen patients with HCC who underwent cisplatin-based TAI chemotherapy (80–100 mg/body) between November 2007 and December 2009 were enrolled in this study. Before administration of cisplatin, granisetron hydrochloride (5-HT3-receptor antagonist) 40 *μ*g/kg and dexamethasone sodium phosphate 8 mg were administered intravenously to all patients. This study was reviewed and approved by the institutional review board of Chiba University School of Medicine and Chiba Cancer Center. Informed consent was obtained from all patients. Age, body mass index (kg/m^2^), performance status scale, clinical UICC TNM stage, Child-Pugh classification, and hepatitis virus test in these patients are listed in Table 1.

### 2.2. Blood Sampling and Measurement of Acylated and Desacyl Ghrelin, Leptin, and Cytokines

Blood samples were obtained before breakfast after an overnight fast before therapy and at day 1, day 7, and day 14 after chemotherapy. The plasma samples were promptly centrifuged at 4°C, and the supernatants were acidified with 1 mol/L HCl (1/10 volume). The ghrelin level was determined using the Active Ghrelin or Desacyl Ghrelin Enzyme-Linked Immunoassay Kit (Mitsubishi Kagaku Iatron, Inc., Tokyo, Japan). Leptin in serum were measured by radioimmune assays. Cytokines in peripheral blood were measured by the Bio-Plex Suspension Array System (Bio-Rad Laboratories, Hercules, CA, USA). Cytokines were as follows: interleukin-1*β*, -1Ra, -2, -4, -5, -6, -7, -8, -10, -12, -13, granulocyte macrophage colony-stimulating factor (GM-CSF), interferon *γ*, tumor necrosis factor alpha (TNF-*α*), granulocyte colony-stimulating factor (G-CSF), monocyte chemotactic protein-1 (MCP-1), and macrophage inflammatory protein-1 beta (MIP-1*β*).

### 2.3. Outcome Measures

Appetite profile was measured using a 100 mm visual analog scale (VAS). Food intake was scored calculated using eleven stages from 0 to 10 by nurses (0 = no intake, 10 = full intake). Adverse events were evaluated by the toxicity grading criteria of Common Terminology Criteria for Adverse Event (CTCAE), version 3.0.

### 2.4. Statistical Analysis

The baseline data are presented as mean ± SD. Wilcoxon signed rank test and Spearman product moment correlation coefficient analysis were used for statistical analyses as appropriate with the statistical program SPSS version 20 (SPSS Inc., Chicago, IL, USA); a *P* value of less than 0.05 was considered statistically significant.

## 3. Results

### 3.1. Changes in Food Intake and Appetite after Chemotherapy

**Figure 1:**
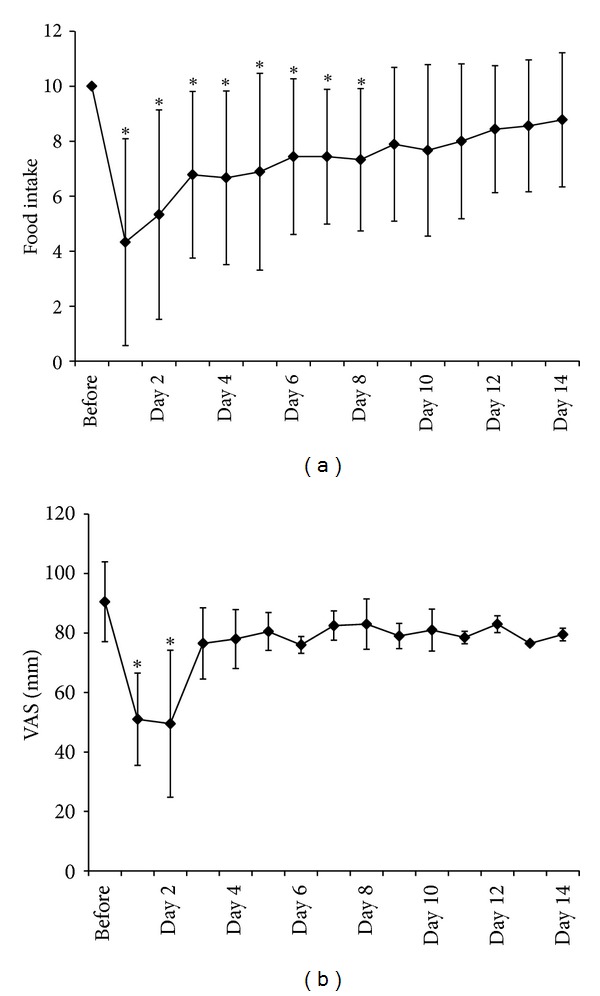
Changes in (a) food intake and (b) visual analog scale (VAS) before and after chemotherapy. Food intake from day 1 to day 8 was significantly decreased compared with prechemotherapy (*P* < 0.05, Wilcoxon signed rank test). Appetite on day 1 and day 2 was also decreased significantly (*P* < 0.05, Wilcoxon signed rank test).

Changes in food intake and appetite are illustrated in Figures 1(a) and 1(b). Food intake scores (0 = no intake, 10 = full intake) before therapy, days 1, 2, 3, 4, 5, 6, 7, and 8 were 10.0, 4.3 ± 3.8, 5.3 ± 3.8, 6.8 ± 3.0, 6.7 ± 3.2, 6.8 ± 3.6, 7.4 ± 2.5, and 7.3 ± 2.9, respectively. Food intake from day 1 to day 8 was significantly decreased compared with prechemotherapy (*P* < 0.05, Wilcoxon signed rank test). Visual analog scales (VASs) of appetite before therapy and day 1, day 2, and day 3 were 76.0 ± 22.9, 56.3 ± 19.0, 57.1 ± 24.1, and 59.0 ± 21.7 mm, respectively. Appetite on day 1 and day 2 was also decreased significantly (*P* < 0.05, Wilcoxon signed rank test). There were no severe adverse events (CTCAE grades 3-4) in this period.

### 3.2. Changes in Plasma Ghrelin Levels after Chemotherapy

**Figure 2:**
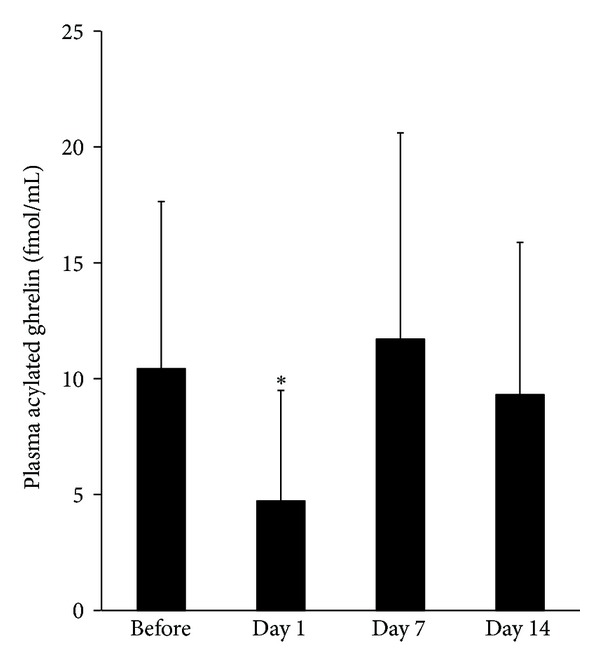
The level of plasma acylated Ghrelin after chemotherapy. The levels of acylated ghrelin were measured in blood samples, which were collected before therapy and at day 1, day 7, and day 14 after chemotherapy. The levels on day 1 were significantly lower than before chemotherapy (*P* < 0.05, Wilcoxon signed rank test).

Changes in plasma acylated ghrelin levels are illustrated in Figure 2, and plasma desacyl ghrelin levels, total ghrelin levels, and the ratios of acylated/desacyl ghrelin (A/D ratio) are summarized in Table 2. Plasma acylated ghrelin levels before therapy, day 1, day 7, and day 14 after chemotherapy were 10.4 ± 7.2, 4.7 ± 4.7, 11.7 ± 8.9, and 9.3 ± 6.6 fmol/mL, respectively. The level on day 1 was significantly lower than before chemotherapy (*P* < 0.05, Wilcoxon signed rank test). However, the fall in plasma ghrelin levels recovered by day 7 to the level of prechemotherapy. The plasma desacyl ghrelin levels, total ghrelin levels and the ratio of A/D did not differ significantly.

### 3.3. Changes in Leptin Levels and Cytokines after Chemotherapy

**Figure 3:**
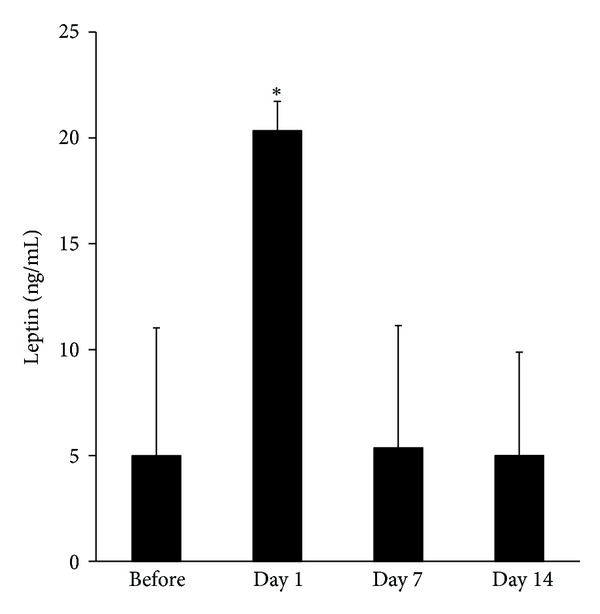
The levels of leptin after chemotherapy. The levels of leptin were measured in blood samples, which were collected before therapy and at day 1, day 7, and day 14 after chemotherapy. The levels of leptin on day 1 were significantly higher than before chemotherapy (*P* < 0.01, Wilcoxon signed rank test).

Changes in leptin levels are illustrated in Figure 3. Leptin levels before therapy and at day 1, day 7, and day 14 after chemotherapy were 5.3 ± 5.6, 20.9 ± 13.8, 7.1 ± 8.4, and 5.2 ± 4.9 ng/mL, respectively. The levels of leptin on day 1 were significantly higher than before chemotherapy (*P* < 0.01, Wilcoxon signed rank test). The levels of IL-12, GCS-F, and MCP-1 in plasma on day 1 were 1.6 ± 0.8, 18.8 ± 10.5, and 99.3 ± 105.4 pg/mL, respectively, which were significantly different (*P* < 0.05, Wilcoxon signed rank test). Other cytokines did not change significantly in this study (Table 3). 

### 3.4. Correlation between Plasma Acylated Ghrelin Levels and Food Intake

To investigate the relationship between peptide hormones and food intake activity, we investigated the correlation between plasma acylated ghrelin levels and food intake. However, there was no correlation between acylated ghrelin levels and food intake (Spearman product moment correlation, *r* = 0.23). In addition, the relationship between leptin levels and food intake did not correlate (Spearman product moment correlation, *r* = − 0.25) in this study. The relationships between Δ acylated ghrelin (change from the levels of pre-chemotherapy) and Δ food intake and between Δ leptin and Δ food intake were also investigated, but there were not correlations, similarly.

## 4. Discussion

Even with the great progress of modern medicine, many patients who undergo chemotherapy still suffer from gastrointestinal disorders. Recently, a number of peptides have been newly discovered and their actions on gastrointestinal (GI) functions have been widely investigated. These new peptides and receptor analogs lead to obtaining therapeutic strategies on the functional disorders in GI tracts. After the discovery of ghrelin, prokinetic effects of ghrelin on functional GI disorders have been widely documented [10–15].

Ghrelin is a 28-amino acid peptide that was discovered in 1999 by Kojima and his colleagues [2] and is known as a potent stimulator of growth hormone release, food intake, and weight gain. Ghrelin was measured in patients with various diseases, for example, breast cancer, colon cancer,and hepatocellular carcinoma. The relationship between the disease and the level of ghrelin was assessed [16–18]. Recently, it has been reported that cisplatin-based chemotherapy reduced plasma ghrelin levels and food intake in rodents [19]. However, only a few investigations in patients with certain types of cancer have been reported [20, 21]. Hiura et al. reported that cisplatin-based chemotherapy reduced plasma ghrelin levels and food intake activity in esophageal cancer patients [21]. In our study, the levels of ghrelin decreased after TAI chemotherapy similarly. In addition, appetite and food intake decreased after chemotherapy in the same way, so we investigated the correlation between plasma acylated-ghrelin levels and food intake. However, the levels of acylated-ghrelin and food intake were not correlated. In addition, the levels of leptin and food intake, similarly, were not correlated. Unfortunately, we could not demonstrate such a direct effect of peptide hormones on food intake after chemotherapy in this study. We need further analysis using a number of patients.

Leptin, a protein mainly produced by adipocytes, acts as a negative feedback signal to the normal control of food intake and body weight [22]. In addition, leptin exerts important regulatory effects on inflammation [23]. In our study, the levels of leptin were significantly increased after chemotherapy. There are some reports about the change in leptin levels after chemotherapy [21, 24]. Tas et al. reported that leptin levels were decreased after chemotherapy in advanced-stage nonsmall cell lung cancer patients. In contrast, Hiura et al. reported that leptin levels were not changed after chemotherapy in esophageal cancer patients. However, they investigated changes after two cycles of chemotherapy [21] and on day 8 after chemotherapy [24]. This difference between our reports and theirs might be caused by the difference in the period of evaluation (acute phase versus later phase after chemotherapy).

In this study on cytokines, the levels of IL-12, MCP-1, and GCS-F were changed after chemotherapy. IL-12 is proinflammatory cytokines that drive the Th1 cell response, characterized by high levels of IFN-*γ* [25]. MCP-1 is chemokine, which has the function of recruiting and activating monocytes/macrophages from circulation to inflammatory sites. G-CSF is a hematopoietic growth factor that stimulates the proliferation and differentiation of neutrophil precursor cells. Recently, ghrelin was reported to have the potential of endogenous anti-inflammatory activities ameliorating some pathologic inflammatory conditions [26, 27]. Zhang et al. reported that exogenous ghrelin could significantly inhibit TNF-*α*/IFN-*γ*-induced CD40 expression in human umbilical vein endothelial cells [28]. Kodama et al. also reported that three-week ghrelin administration decreased neutrophil density and inflammatory cytokine levels (TNF-*α*, IL-8) in sputum [29]. In this study, the levels of ghrelin were changed but the levels of TNF-*α*, IFN-*γ*, and IL-8 were not changed; instead of this, IL-12, MCP-1, and GCS-F were changed after chemotherapy. These changes might be caused by the decrease of ghrelin or by the direct effect of chemotherapy. However, the relationship between such cytokines and chemotherapy remains unclear.

Finally, in the near feature, administration of ghrelin might be a significant help for patients who suffer from gastrointestinal disorders after chemotherapy. In fact, there are some reports about the effect of intravenous administration of ghrelin in humans [9]. On the other hand, there are some reports of oral medication which affect secretion of ghrelin [8, 30]. We reported that traditional Japanese medicine Rikkunshito increases the plasma levels of ghrelin in humans and mice [30]. This oral medication might alleviate the suffering of patients, caused by chemotherapy.

In conclusion, TAI for HCC reduced plasma acylated-ghrelin levels and food intake significantly. In addition, it increased serum leptin levels significantly. This is the first report showing that transcatheter arterial infusion chemotherapy, which is different from systemic chemotherapy, reduces plasma ghrelin levels and food intake.

## Figures and Tables

**Table 1:** Patient characteristics.

	*n* = 17
Sex (M/F)	15/2
Age (years, ± SD)	69.2 ± 7.1
BMI (kg/m^2^, ± SD)	24.2 ± 3.3
Performance status scale (0/1/2/3/4)	16/1/0/0/0
Hepatitis virus (HBV/HCV/both negative)	3/12/2
Clinical UICC TNM stage (I/II/IIIA/IIIB/IIIC/IV)	0/6/5/1/1/4
Child-Pugh classification (A/B/C)	12/5/0

BMI: body mass index. HBV: hepatitis B virus. HCV: hepatitis C virus.

**Table 2:** The levels of plasma acylated ghrelin, desacyl ghrelin, total ghrelin, and A/D ratios before and after chemotherapy.

	Acylated ghrelin	Desacyl ghrelin	Total ghrelin	A/D ratio
Before	10.4 ± 7.2	183.7 ± 213.8	180.4 ± 212.4	0.08 ± 0.06
Day 1	4.7 ± 4.7*	119.3 ± 170.1	127.1 ± 167.4	0.11 ± 0.10
Day 7	11.7 ± 8.9	137.2 ± 152.4	125.1 ± 147.1	0.12 ± 0.08
Day 14	9.3 ± 6.6	143.3 ± 162.1	141.8 ± 163.9	0.11 ± 0.02

*P < 0.05. Wilcoxon signed rank test compared with prechemotherapy. A/D ratio: ratio of acylated/desacyl ghrelin.

**Table 3:** The levels of leptin and cytokines.

	Before	Day 1	Day 7	Day 14
Leptin (ng/mL)	4.6 ± 5.1	20.9 ± 13.8*	7.1 ± 8.4	5.2 ± 4.9
IL-1*β* (pg/mL)	1.5 ± 1.7	1.7 ± 2.7	1.5 ± 1.3	1.1 ± 1.2
IL-1Ra (pg/mL)	325.6 ± 383.8	371.9 ± 210.4	268.9 ± 275.5	415.0 ± 460.7
IL-2 (pg/mL)	21.7 ± 16.6	18.2 ± 18.6	26.9 ± 18.8	22.1 ± 23.9
IL-4 (pg/mL)	0.4 ± 0.5	—	0.4 ± 0.3	0.3 ± 0.3
IL-5 (pg/mL)	1.2 ± 0.9	102.2 ± 204.0	2.8 ± 3.9	1.1 ± 0.6
IL-6 (pg/mL)	12.5 ± 21.7	86.1 ± 116.4	18.4 ± 14.1	40.6 ± 96.7
IL-7 (pg/mL)	3.2 ± 3.3	1.8 ± 0.9	2.9 ± 2.3	2.9 ± 3.3
IL-8 (pg/mL)	18.9 ± 22.3	33.9 ± 28.8	25.8 ± 30.5*	27.0 ± 32.2
IL-10 (pg/mL)	1.7 ± 2.7	5.1 ± 5.1	1.8 ± 1.7	3.1 ± 3.1
IL-12 (pg/mL)	25.9 ± 92.0	1.6 ± 0.8*	2.6 ± 2.4	3.5 ± 3.1
IL-13 (pg/mL)	2.1 ± 1.7	3.9 ± 4.8	2.1 ± 1.5	1.9 ± 1.6
GM-CSF (pg/mL)	16.8 ± 27.1	49.3 ± 60.1	23.8 ± 35.9	2.4 ± 2.6
IFN-*γ* (pg/mL)	15.1 ± 20.8	11.8 ± 23.8	12.1 ± 19.3	8.7 ± 8.7
TNF-*α* (pg/mL)	10.9 ± 25.6	15.4 ± 38.6	10.9 ± 27.6	8.2 ± 12.2
G-CSF (pg/mL)	8.8 ± 7.2	18.8 ± 10.5*	9.1 ± 5.8	8.8 ± 3.7
MCP-1 (pg/mL)	33.6 ± 14.2	99.3 ± 105.4*	28.8 ± 16.9	41.9 ± 40.0
MIP-1*β* (pg/mL)	86.7 ± 26.2	168.2 ± 167.3	91.7 ± 26.3	138.4 ± 212.5

*P < 0.05. Wilcoxon signed rank test compared with prechemotherapy. GM-CSF: granulocyte macrophage colony-stimulating factor. G-CSF: granulocyte-colony-stimulating factor. TNF: tumor necrosis factor. MCP-1: monocyte chemotactic protein-1. MIP-1*β*: macrophage inflammatory protein-1*β*.
